# Advances in Deubiquitinating Enzyme Inhibition and Applications in Cancer Therapeutics

**DOI:** 10.3390/cancers12061579

**Published:** 2020-06-15

**Authors:** Ainsley Mike Antao, Apoorvi Tyagi, Kye-Seong Kim, Suresh Ramakrishna

**Affiliations:** 1 Graduate School of Biomedical Science and Engineering, Hanyang University, Seoul 04763, Korea; ainsleyantao@gmail.com (A.M.A.); apoorvityagi09@gmail.com (A.T.); 2 College of Medicine, Hanyang University, Seoul 04763, Korea

**Keywords:** cancer therapy, deubiquitinase, DUB inhibitors, USPs phylogenetic tree, signaling pathways

## Abstract

Since the discovery of the ubiquitin proteasome system (UPS), the roles of ubiquitinating and deubiquitinating enzymes (DUBs) have been widely elucidated. The ubiquitination of proteins regulates many aspects of cellular functions such as protein degradation and localization, and also modifies protein-protein interactions. DUBs cleave the attached ubiquitin moieties from substrates and thereby reverse the process of ubiquitination. The dysregulation of these two paramount pathways has been implicated in numerous diseases, including cancer. Attempts are being made to identify inhibitors of ubiquitin E3 ligases and DUBs that potentially have clinical implications in cancer, making them an important target in the pharmaceutical industry. Therefore, studies in medicine are currently focused on the pharmacological disruption of DUB activity as a rationale to specifically target cancer-causing protein aberrations. Here, we briefly discuss the pathophysiological and physiological roles of DUBs in key cancer-related pathways. We also discuss the clinical applications of promising DUB inhibitors that may contribute to the development of DUBs as key therapeutic targets in the future.

## 1. Introduction

Cancer is fundamentally a genetic disorder that has been well characterized over the past decade. The genetic basis of different cancers has been investigated thoroughly with the application of high-throughput genome-wide association studies (GWAS), which have identified over 450 genetic variations associated with cancer risk [1]. These mutations cause cancer susceptibility, not cancer per se, providing a “head start” to the neoplastic process [2]. Germline or somatic mutations in three major classes of genes, oncogenes, tumor suppressor genes, and DNA stability genes, initiate the neoplastic process, resulting in rounds of clonal expansion and subsequent somatic mutations associated with tumor formation [3].

Proto-oncogenes are required for normal cellular homeostasis and functions. Mutations in proto-oncogenes resulting from chromosomal translocations or gene fusions spark constitutive expression under conditions where wild-type genes would be inactive [4,5]. Cancer cells, therefore, gain competitive advantages over their non-neoplastic counterparts, while mutations in tumor suppressor genes confer neoplastic cells with competitive advantages in an opposite way. Tumor suppressor genes function to control tumor formation by the stimulation of cell death and restrain inappropriate cell division. The inactivation of tumor suppressor genes arises from missense mutations or indels at active site residues, resulting in truncated proteins or epigenetic silencing [6]. A subset of tumor suppressor genes, called caretaker genes or DNA stability genes, maintain genetic integrity through nucleotide-excision repair, mismatch repair, base-excision repair, chromosomal segregation, and mitotic recombination [7,8]. Because they encode molecules that stabilize the genome, mutations in caretaker genes can contribute to the neoplastic process. Although the accumulation of several gene mutations contributes to fully fledged cancer, a few targeted pathways are involved. Examples of these, such as receptor tyrosine kinase (RTKs) signaling and the p53 pathway, will be discussed in this review [9,10]. 

Other important regulators of cancer progression are cellular proteases, which play indispensable roles in various biological and pathological processes [11,12]. Proteases are highly specific enzymes that selectively carry out proteolytic processing, targeting a wide range of substrates, thereby regulating processes essential for cell survival [13]. A deficiency of proteases or misdirected spatio-temporal expression patterns can cause diverse pathologies, such as neurodegenerative diseases, arthritis, cardiovascular diseases, and cancers, making them an important focus as drug targets or diagnostic markers [13,14]. The entire repertoire of cellular proteases, called the “degradome,” comprise about 588 proteases divided among five catalytic classes known as the aspartyl, cysteine, metallo-, serine, and threonine proteases [15]. Most of these proteolytic enzymes catalyze the hydrolysis of peptides by targeting the α-peptide bonds between naturally occurring amino acids, while others undertake slightly different reactions. Among them lie a growing group of peptidases known as deubiquitinating enzymes (DUBs) that hydrolyze isopeptide bonds in ubiquitin and ubiquitin-like protein conjugates and, thus, have emerged as crucial regulators of ubiquitin-mediated signaling pathways and are potential drug targets in various diseases, including cancer [16,17,18]. 

## 2. Ubiquitin Proteasomal Pathways and DUBs

Ubiquitination is a very important post-translational modification that plays multifaceted roles in cancer-related pathways. The ubiquitination process is also involved in protein metabolism, apoptosis, cell-cycle progression, and transcription. It involves the covalent attachment of a 76-amino acid protein called ubiquitin (Ub) to the ε-amino groups of lysine residues in the target proteins. This conjugation reaction is catalyzed by the successive action of three enzymes: E1 or Ub-activating enzyme, E2 or Ub-conjugating enzyme, and E3 or Ub-ligase enzyme [19]. The first step of this process is the ATP-dependent activation of the Ub molecule by E1, forming a thioester bond with the C-terminal of Ub. The activated Ub is then transferred to E2, forming a thioester-linked E2-Ub intermediate, which is then positioned and transferred to the target substrate protein with the help of E3. Ub can be conjugated as monomers or multiple mono-ubiquitin adducts to either the same or different residues of the target protein that directs their fate. Lysine 48 (K48), along with K11- and K29-linked poly-ubiquitin chains, targets protein destruction via the 26S proteasome. However, K63-linked poly-ubiquitination and multiple mono-ubiquitin conjugations are mainly involved in proteasome-independent events such as DNA repair and signal transduction [20,21,22], although their involvement in proteasomal targeting has also been reported [23].

Interestingly, protein ubiquitination is a dynamic and reversible process. DUBs are responsible for the removal of ubiquitin from their target proteins, rescuing them from the degradative pathway. They are also involved in the editing, maturation, and recycling of the ubiquitin molecule post degradation [24]. Some DUBs can edit the Ub signal harbored by target proteins by trimming Ub chains. For example, A20 causes K48-linked proteasomal degradation instead of K63-linked polyubiquitination of receptor-interacting serine-threonine kinase 1 (RIPK1) [25]. DUBs are highly versatile and capable of preventing lysosomal as well as proteasomal degradative pathways, thus enhancing protein stability. The deubiquitination process maintains the pool of free mono-Ub that is recycled for the ubiquitination of misfolded proteins and is therefore important in maintaining the normal rates of proteolysis within cells. Moreover, DUBs prevent the attachment of inhibitory ubiquitin chains that compete with ubiquitinated protein substrates at the binding sites of the 26S proteasome. In a nutshell, DUBs maintain the stability of protein substrates and regulate various cellular processes, such as epistasis, gene expression, chromosome segregation, DNA repair, spermatogenesis, signaling events, and cell cycle regulation [16,26]. 

**Figure 1:**
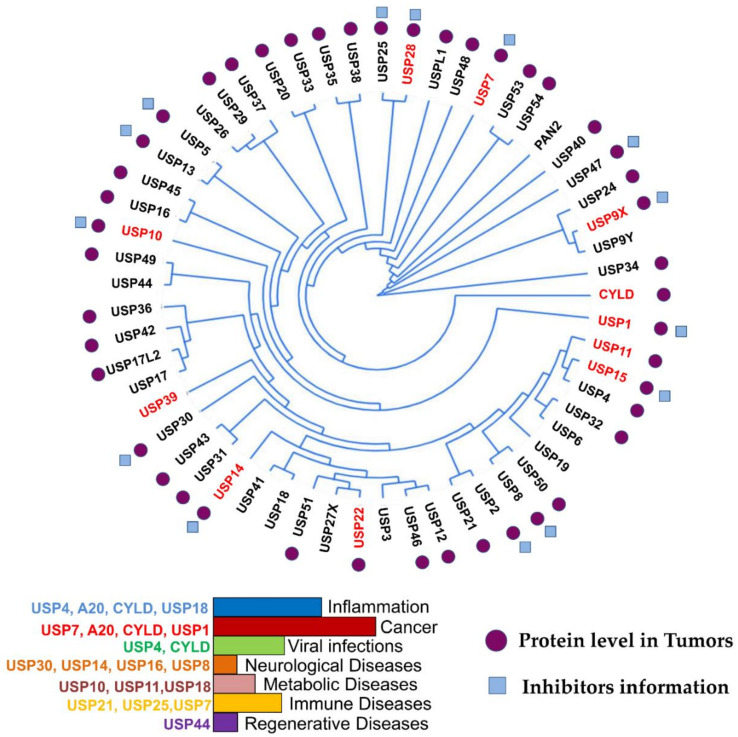
USP phylogenetic tree. Ubiquitin-specific proteases (USPs) and their association with different diseases are indicated in the histogram. The branches arising from one node represent a clade. The distance between the USPs in the phylogenetic tree describes the relationship among the individuals in this family. DUBs with high expression in different cancers are highlighted in red in the phylogenetic tree. Tumors expressing medium levels of USPs in different cancer types are indicated with purple circles. Blue boxes indicate USPs with identified drugs. This figure is a representation of data obtained from at least 1% of cancer patients [30].

More than 100 functional DUBs have been identified across the human genome and are categorized into eight families: ubiquitin-specific proteases (USPs), ubiquitin C-terminal hydrolases (UCHs), ovarian tumor proteases (OTUs), Jab1/MPN domain-associated metallopeptidase (JAMM) domain proteins, Josephin or Machado–Joseph disease protein domain proteases (MJDs), the monocyte chemotactic protein-induced protein (MCPIP) family, the motif interacting with Ub-containing novel DUB family (MINDY), and Zn-finger and UFSP domain proteins (ZUFSPs) [16,27,28,29]. Figure 1 presents the phylogeny, role in cancer and other diseases, and inhibitors of fourteen USPs, the largest subfamily of DUBs. 

DUBs control several aspects of cell physiology, and defects in these processes have many clinical implications. Examples of these deregulations include the BRCA1-associated protein 1 (BAP1) of the UCH family of DUBs, which is commonly mutated in mesotheliomas, melanomas, and renal cell carcinomas [31]; USP6 translocation in aneurysmal bone cysts [32]; USP9X mutations that cause developmental disorders and deregulated expression in cancer [33,34]; USP15 overexpression in certain glioblastomas, ovarian cancer, and breast cancer [35]; and cylindromatosis (CYLD) mutations in familial cylindromatosis [36]. Due to the involvement of the components of ubiquitin machinery in different cancer types, inhibitors targeting DUBs are attracting much attention from pharmaceutical industries, and several candidates have already been identified as potentially rewarding drug targets. This review focuses on the production of chemical libraries consisting of inhibitors that specifically target DUBs involved in cancer.

## 3. DUBs in Cancer

The functions of DUBs are not just limited to the reversal of ubiquitination but encompass multi-dimensional aspects like protein trafficking, apoptosis, chromatin remodeling, DNA damage repair, cell cycle regulation, and signaling pathway modulations that are frequently linked to the development of neoplastic diseases [17,37]. DUBs have been implicated in tumorigenesis at multiple levels, and, after the clinical success of the broadly acting proteasome inhibitor bortezomib, used to treat refractory multiple myelomas (MM) and myeloid cell leukemia (MCL), DUBs have become attractive targets for the development of targeted novel therapies in cancer [38,39]. DUB modifications and their implications in different cancers are described briefly here to gain insight into their role as potential drug targets. 

### 3.1. Genetically Altered DUBs in Cancer 

Genetically altered DUBs have been identified in cancers, corroborating their roles as true oncogenes and tumor suppressors. Several studies implicate USP4, USP7, USP6, USP15, USP16, USP42, and USP28 as oncogenes, whereas CYLD, A20, and BAP1 act as tumor suppressors [37,40,41,42]. There are reports that DUBs such as USP7, CYLD, and A20 play dual roles in cancer, acting as both anti- and pro-tumorigenic enzymes according to the affected targets [43,44,45]. USP6, or Tre2, acts as an oncogene due to chromosomal rearrangements of the osteoblast cadherin11 gene (CDH11) promoter, which cause an overexpression of USP6 linked to neoplastic aneurismal bone cysts [46]. USP6 also regulates expression of the proto-oncogene c-Jun and promotes cell invasion [47]. Somatic mutations in the USP28 gene have been identified in lobular breast cancer cases [48]. 

CYLD mutations have been described in familial cylindromatosis, familial trichoepithelioma, and Brooke–Spiegler syndrome, in which patients have a predisposition to develop multiple skin tumors of the neck and head [49]. Nuclear factor (NF)-κB signaling has well-established roles in cancer promotion and CYLD has been reported to negatively regulate this pathway through its deubiquitinating activity [50,51,52,53]. Several lymphoma subtypes show chromosomal deletions and inactivating mutations in the A20 gene loci. Inactivating A20 mutations have been reported in marginal zone lymphomas [54] and A20 mRNA expression loss has been found in non-Hodgkin’s lymphoma, Burkitt’s lymphoma, and T-cell lymphoma [55]. A20, also called tumor necrosis factor alpha-induced protein 3 (TNFAIP3), is a negative NF-κB signaling regulator [56]. Innate immune signals, including the tumor necrosis factors and toll-like receptors, trigger A20-mediated termination of NF-κB signaling. 

BAP1, or BRCA1-associated protein, is a nuclear-localized DUB and may be the most commonly mutated DUB in cancers. Originally identified as an interacting partner of the BRCA1 tumor suppressor, BAP1 augments tumor suppressor functions but has not been identified as a DUB for BRCA1 [57]. Germline mutations in BAP1 are associated with tumor predisposition syndrome for mesotheliomas and melanocytic tumors [58,59], whereas deletions and point mutations have been identified in lung and breast cancers [57,60,61]. BAP1-inactivating mutations are also identified in metastasizing uveal melanomas and malignant pleural mesotheliomas [60,62]. BAP1 also controls cell cycle progression by the regulation of host cell factor-1 (HCF-1) and other G1-S-transition-related genes [63].

### 3.2. DUBs Regulating Signaling Pathways in Cancer

Signaling pathways are receiving more attention than the individual genes that are altered in cancers [3,64]. DUBs have a profound influence over commonly mutated cancer pathways such as p53, NF-κB, RTKs, Wnt, and transforming growth factor (TGF)-β, which are briefly described below.

#### 3.2.1. p53 Signaling 

The tumor suppressor p53 is lost or mutated across most tumor types [65]. p53 has evolved an exquisite regulatory mechanism enabling rapid stress responses and preventing errant activation, mainly due to the pivotal role that it plays in cellular activities [66]. The p53 inhibitors ARF-BP1, MDM2, Pirh2, COP1, and MDM4 of the E3 ubiquitin ligase family are central to this regulation [67]. Several DUBs have been identified as important p53 regulators. Thus far, USP2a, USP4, USP5, USP7, USP9X, USP10, USP11, USP15, USP24, USP29, and USP49 have been associated with p53 regulation [68]. USP2 and its isoforms have previously been shown to have oncogenic properties and have been extensively investigated by cancer biologists. USP2a has high expression levels in human prostate adenocarcinomas, and treatment with chemotherapeutic agents prevents the apoptosis of cancerous cells [69,70,71]. USP2 has been reported to deubiquitinate proteins involved in different pathways, such as MDM2, MDMX, apoptosis inducing factor (AIF), Cyclin D, fatty acid synthase (FASN), Aurora-A kinase, and epidermal growth factor receptor (EGFR) [72,73,74,75,76,77,78]. USP2a does not directly deubiquitinate p53 but acts on MDM2 without reducing p53 ubiquitination. MDM2 is an E3 ligase for p53 and promotes its ubiquitination and degradation; therefore, suppression of USP2a leads to the activation of p53 in vivo [73]. However, in glioma cells, USP2a binds to MDM4, enhancing the localization of p53 to the mitochondria and promoting apoptosis [79]. Stabilization of ARF-BP1 via USP4 deubiquitination has been reported to reduce p53 levels [66]. Enhanced apoptosis in thymus and spleen has been observed in USP4 knockout mice in response to ionizing radiation. USP4^-/-^ mouse epithelial fibroblasts (MEFs) exhibit premature cellular senescence, retarded growth, resistance to oncogenic transformation, and hyperactive DNA damage checkpoints compared to wild-type MEFs. These observations indicate higher p53 levels and activity due to the absence of USP4. USP4 has been suggested as a potential oncogene and is observed to be highly upregulated in several cancers [66].

USP5 negatively regulates the level and transcriptional activity of p53. USP5 disassembles unanchored poly-ubiquitin chains from its substrate to recycle free mono-ubiquitin and contributes to K48-linked poly-ubiquitin disassembly [80]. Suppression of USP5 inhibits p53 proteasomal degradation without affecting MDM2, ultimately resulting in p53 activation. USP5 is, therefore, a potential p53-activating therapeutic drug target in cancer therapy [80]. USP7 dynamically regulates p53 as well as MDM2 [45,81]. Inhibition of USP7 triggers MDM2 degradation and p53 stabilization, ultimately leading to apoptotic pathway activation in tumor cells [82]. USP7 inhibitors can have alternative therapeutic mechanisms of action, as USP7 has several other targets including forkhead box protein O4 (FOXO4), FOXP3, phosphatase and tensin homolog (PTEN), and UBL SUMO [83,84,85,86]. USP10 is identified as a cytoplasmic DUB that regulates p53 levels and localization by reversing Mdm2-induced p53 nuclear export and degradation. It regulates the stability of p53 without interacting with MDM2, unlike USP2 and USP7, and has been shown to stabilize the wild type as well as the mutated form of p53, playing a dual role in tumorigenesis, which depends upon the status of p53 [87]. USP28 is highly expressed in many cancer types, such as in breast cancer [88], non-small-cell lung cancer (NSCLC) [89], gastric cancer [90], bladder cancer [91], and colorectal cancer [92]. USP28 is an indirect regulator of the proto-oncogene c-Myc via E3 ligase Fbw7a as well as JUN and Notch [93]. USP28 is also found to regulate lysine-specific demethylase 1 (LSD1) protein levels in gastric cancer cells and has also been connected to p53 via TP53-binding protein 1 (TP53BP1) [88,94]. Due to the role played by USP28 in various malignancies, it is identified as a potential drug target for cancer treatments. In response to oxidative stress, USP29 deubiquitinates and stabilizes p53 by reversing MDM2-directed p53 ubiquitination [95]. 

USP42 was identified as a p53 DUB by siRNA library screening and deubiquitinates p53 by removing Mdm2-conjugated Ub [96]. It stabilizes p53 and helps recruit p53-interacting factors contributing to p53 functions [97]. USP9X attenuates the degradation of p53 and controls several cellular functions, including proliferation, apoptosis, and chemo-resistance. Studies have shown that USP9X mediates apoptosis and/or survival in p53-deficient cells [98]. USP24 regulates p53 and the apoptosis pathway related to p53 function in the DNA damage response [99]. USP15 upregulates transcriptional activation and the stability of p53 in response to TGF-β signaling, thereby affecting the stability of both Mdm2 and p53 [100]. USP11 stabilizes and activates p53 following DNA damage by targeting ubiquitinated p53. Post DNA damage, USP11 activates p53 and its target genes such as *Puma, Bax*, and *p21* [101]. USP49 has also been reported as a novel positive regulator of p53 transcriptional activity and stability. It has been shown to render HCT119 cells more sensitive to DNA damage induced by etoposide and is upregulated in response to DNA damage and other cell stresses, thus acting as a potential tumor suppressor and forming a positive feedback loop with p53 [102].

#### 3.2.2. Nuclear Factor-κB Signaling

The NF-κB transcription factor family members have been recognized as crucial players in cancer initiation and progression while also playing roles in inflammation and innate immunity [103]. A20 and CYLD act on several components of the pathway, downregulating NF-κB signaling and thereby acting as tumor suppressors [104]. Several intermediates of the NF-κB signaling pathway, NEMO, TRAF-2, and TRAF-6, undergo K63 poly-ubiquitination post-receptor activation and activate the IκB kinase (IKK). IKK, in turn, phosphorylates NF-κB inhibitor IκB, leading to its dissociation from NF-κB, K48-linked poly-ubiquitination by the phospho-specific E3 ligase β-TrCP, and finally its proteasomal degradation [51,52,53,105]. CYLD potentially dampens NF-κB signaling following the removal of K63 poly-ubiquitin chains from TRAF-2, TRAF-6, and NEMO. The generation of mutant CYLD^-/-^ mice has confirmed the role of this DUB as a tumor suppressor. The ability of CYLD to prevent cell proliferation was observed by the effect on skin tumor formation. A single 12-O-tetradecanoylphorbol-13 acetate (TPA) application resulted in an increase in Ki-67 and cyclin D1 in skin sections of CYLD^-/-^ animals [106]. Colon tumor induction in CYLD^-/-^ mice has been linked to inflammation associated with enhanced NF-κB activity [107]. These studies collectively strengthen the role of CYLD as an important tumor suppressor in humans.

A20 regulates NF-κB pathways through its ovarian tumor deubiquitinase (OTU) domain and zinc-finger domain [108,109]. The OTU domain is responsible for deubiquitinating NF-κB intermediates such as IKKg, RIP1, RIP2, TRAF2, and TRAF6, and shows a stronger preference for K63-linked Ub chains. The zinc-finger domain is responsible for the Ub ligase activity of A20, replacing the K63-linked chains with proteasome-targeting K48 chains [110,111]. USP21 is another DUB identified as an inhibitor of the NF-κB pathway, which interacts and deubiquitinates RIP1 in a DUB-dependent manner [112]. USP4 has an important role in the downregulation of TNFα-induced activation of NF-κB via TGF-β-activated kinase 1 deubiquitination [113]. Cezanne interacts with DJ-1 and targets RIPK1 signaling intermediates, thereby suppressing NF-κB nuclear translocation and its activity [114,115]. USP31 deubiquitinates K63-linked poly-ubiquitinated proteins of the TNF-induced NF-κB pathway [116]. USP7 deubiquitinates TRAF6 and NEMO, thus negatively regulating NF-κB in the TLR pathway while USP2 acts as a positive regulator of TNF-induced NF-κB activation [117,118]. USP2 is required for IκB phosphorylation and the nuclear translocation of NF-κB [118]. OTUD5 targets TRAF3 for deubiquitination, resulting in diminished IL-10 and type I interferon responses [119]. USP15 stabilizes IκBα, whereas USP11 interacts with the inhibitor IKKα upon TNFα induction [120,121]. MCP-induced protein 1 (MCPIP1) is a novel DUB that targets TRAF2 and TRAF6, which are essential for termination of NF-κB and JNK signaling [29].

#### 3.2.3. Receptor Tyrosine Kinase Signaling

Growth factor receptors are composed mainly of transmembrane, extracellular, and cytoplasmic tyrosine kinase (TK) domains. RTK activation is required for the regulation of key processes, such as cell growth, survival, tissue repair, and regeneration; therefore, dysregulation of this pathway has been reported in numerous cancers. The significance of the RTK pathway makes it an attractive therapeutic target [122]. The mechanism responsible for the delivery of membrane receptors from the cell surface to lysosomes has been intensively studied for EGFR, and ubiquitination serves as a key signal for this pathway. The trafficking of RTKs such as Met, ErbB2, and EGFR is regulated by DUBs [123]. USP8 stabilizes RTKs by deubiquitinating EGFR on early endosomes, thus allowing their recycling to the plasma membrane [124]. USP8 has also been reported to promote RTK degradation [125]. RNA interference screens have identified USP18 as an EGFR synthesis regulator and POH1 as an ErbB2 regulator [126,127]. USP18 regulates the expression of EGFR and is responsible for cancer cell survival through mRNA stabilization and transcriptional activation of miR-7 host genes [128]. USP9X is a DUB for Eps15, which is essential for EGFR internalization, thereby acting as an EGFR regulator [129]. Cezanne-1 was identified as an EGFR-regulating DUB using RNA interference screening and is reported to be overexpressed in breast cancer and in approximately one-third of mammary tumors. In conclusion, oncogenic growth signals are promoted following deubiquitination by Cezanne-1, which curtails the degradation of growth factor receptors [130].

#### 3.2.4. Wnt Signaling

Another frequently altered pathway in cancer is the Wnt signaling pathway, which essentially controls embryonic development [131]. Malignant transformation, tumor progression, and resistance to conventional cancer therapy are favored by dysregulation of canonical and non-canonical Wnt-planar cell polarity (PCP) signaling [132,133]. Aberrant Wnt signaling is also suggested to subvert cancer immune surveillance, promoting immune evasion and resistance to immunotherapies and immune checkpoint blockers [134,135,136]. Regulation by ubiquitination and deubiquitination play key roles in canonical Wnt signaling and in non-canonical Wnt-PCP signaling pathways. The ubiquitin-mediated system regulates cytoplasmic β-catenin as well as other proteins upstream of the Wnt pathway [137,138]. USP9X has been reported as a DUB for DVL2, which is required for the activation of canonical Wnt. Increased DVL2 ubiquitination activates the PCP pathway via localization to actin-rich projections, making USP9X an important therapeutic target for cancers [139]. A strong correlation has been found between the levels of β-catenin and USP14 by tissue microarray analysis of colon cancers, suggesting an oncogenic role of USP14 by the enhancement of Wnt/β-catenin signaling [140]. USP6 was identified by genome-wide siRNA screening as a potent activator of Wnt signaling by deubiquitinating Fzds, thereby increasing their cell surface abundance [141]. USP4 is a potential cancer therapy target as it deubiquitinates and facilitates the nuclear localization of β-catenin, acting as a positive regulator of the Wnt/β-catenin pathway [142]. USP34 hinders β-catenin-dependent transcription, acting as a positive Wnt signaling regulator [143].

#### 3.2.5. Transforming Growth Factor-β Signaling

The multifunctional protein TGF-β plays a dual role in oncogenesis, acting as an anti-proliferative factor during the early stages and as an epithelial-to-mesenchymal transition (EMT)-promoting factor in the later stages [144]. So far, only a few DUBs have been reported to regulate this pathway. USP9X acts as a DUB for SMAD4, promoting its association with SMAD2 and positively regulating TGF-β [145]. USP9X also deubiquitinates NAUK1 and MARK4, AMPK-related kinases important for the regulation of cell proliferation and polarity, by modulating their phosphorylation and activation by LKB1 [146]. USP15, thought to be important for the proliferation of glioblastoma cells, deubiquitinates the type I TGFβ receptor via binding to the SMAD7-SMAD-specific E3 ubiquitin ligase 2 (SMURF2) complex [35]. Overexpression of USP15, which correlates with higher TGF-β activity, is mostly found in ovarian cancer, breast cancer, and glioblastomas [35]. Reducing TGF-β signaling by depleting USP15 reduces the oncogenicity of patient-derived glioma-initiating cells, indicating the therapeutic capabilities of USP15 inhibition [147]. USP15 has also been reported to deubiquitinate other TGF-β signaling components and receptor-regulated SMADs (R-SMAD), whereas UCHL5 and AMSH-LP potentiate TGF-β responses through I-SMADs interactions [147,148,149].

### 3.3. DUBs in Cell Cycle Regulation 

The regulation of proteins such as cyclins, cyclin-dependent kinases (CDKs), and other checkpoint molecules that mediate the cell cycle is highly important to ensure proper division of cells. Inappropriate expression or deregulation of these proteins can result in different types of tumors. Several studies have reported the role of DUBs in regulating cell cycle proteins. DUBs such as CYLD, USP5, USP13, USP15, USP17, USP37, USP39, and USP44 are key regulators of events that occur during mitosis [41]. CYLD, USP15, and USP44 modulate spindle formation [41]. USP44 deubiquitinates anaphase-promoting complex (APC) coactivator Cdc20 and prevents chromosomal segregation errors [150]. Mutations in USP44 result in the formation of tumors, especially in lung cancer [150]. USP37 promotes entry into the S phase of the cell cycle by anaphase-promoting complex (APC) coactivator Cdh1 [151]. USP15 regulates the cell cycle by stabilizing and rescuing the expression of newly synthesized REST at mitotic exit [152]. USP17 is tightly controlled in the cell cycle and its knockdown attenuates GTPase signaling and proliferation of tumor-derived cell lines [153]. USP39 is important for the integrity of the mitotic spindle checkpoint, and depleting USP39 during mRNA processing reduces mRNA levels of Aurora B [154]. CYLD regulates polo-like kinase 1 (Plk1) and regulates mitotic entry [155]. USP3 is required for chromatin modification and S phase progression [156]. USP2 prevents ubiquitin-mediated degradation of cyclin-D1. USP2a deubiquitinates and stabilizes the cell cycle regulator Cyclin A1, controlling cell proliferation [70].

## 4. Nuclear Functions of DUBs

DUBs regulate signaling pathways that culminate in changes to gene transcription via specific transcription factors altering stability, activity, and subcellular localizations. Besides these paramount functions, DUBs also influence chromatin structures and co-ordinate DNA repair pathways. The entire cellular genome is tightly packaged in nucleosomes, the basic units of chromatin, which undergo remodeling to accommodate DNA transcription, replication, and cell division. Two major histone proteins, H2A and H2B, commonly undergo post-translational modifications via the deubiquitinating pathways that regulate chromatin structure dynamics and gene transcription. These processes are frequently altered in different types of cancers [157]. DUBs such as USP3, USP22, and USP46 have been reported to act on both H2A and H2B, while MYSM1, USP16, USP21, and BAP1 act on the mono-ubiquitinated H2A, and the K63-specific DUB BRCC3 shows specificity for di-ubiquitinated H2A [158,159,160,161]. USP10 has been reported to specifically act on the H2A.Z variant [162]. USP7 is associated with several factors linked to transcription, and, along with USP11, forms a part of the E3 ligase protein regulator of cytokinesis 1 (PRC1) complex, where they function to stabilize PRC1 components by their DUB activities promoting H2A ubiquitination [163]. DUBs also influence RNA-associated proteins; for example, USP4 has been reported in mRNA splicing [164,165].

The DNA damage response (DDR) is a vital aspect of physiological processes such as homeostasis and cancer prevention and is responsible for cell cycle checkpoint activation, ensuring effective DNA damage repair [166]. Cancer development has been linked to genetic damage repair, as observed in Fanconi anemia, where increased tumor rates occur in diseases with deficient DNA repair mechanisms. USP1 has been reported as a major DUB that regulates multiple aspects of the DDR pathways, including FANCD2, CHEK1, PCNA, and DDB1 [167,168,169,170]. DDRs coordinated by ATM- and ATR-CHEK are also regulated by DUBs including USP15, USP19, USP28, and USP34, of which USP28 is reported to act on CHEK2 in response to double-strand breaks (DSBs) [171,172,173]. Several other DUBs, such as USP3, USP16, BRCC36, and OTUB1, have been implicated in the regulation of DNA repair via the RNF8/168 pathway for DSB repair [174]. USP9X is another potentially important therapeutic DUB that functions to maintain DNA replication fork stability and mediate responses at DNA damage checkpoints via claspin regulation [34,175]. It also affects radio-sensitivity in glioblastoma cells through MCL1-dependent as well as independent mechanisms [176]. 

## 5. Drugs Targeting DUBs in Cancer

As discussed above, the significance of DUBs as key regulators in multiple biological pathways, especially their roles in cancer, has made them attractive targets for the development of new anti-cancer strategies. The DUB inhibitors that have been developed are discussed in the upcoming section in brief and summarized in Table 1.

### 5.1. DUB Inhibition by Compounds Containing Michael Acceptors

Michael-acceptor-containing compounds have inhibitory effects on cysteine DUBs, as they can potentially form covalent adducts with free thiols in active sites [235]. Chalcone compounds and cyclopentenone prostaglandins (PGs) of the PGJ2 class are examples of Michael-acceptor-containing compounds that are discussed below.

b-AP15: USP14 and UCHL5 are highly expressed in MM cells when compared to normal plasma cells. b-AP15 has been identified as a 19S regulatory particle (RP) inhibitor that can selectively block USP14 and UCHL5 activities without proteasomal activity inhibition [177,178]. b-AP15 has been shown to decrease the viability of patient MM cells as well as MM cell lines and, even in the presence of bone marrow stromal cells, inhibits MM cell proliferation and overcomes bortezomib resistance [177]. b-AP15 was also found to induce apoptosis in ovarian cancer cell lines by inhibiting UCHL5 and suppressing *TP53*-mutants, thereby regulating TGF-β signaling [178].

VLX1570: VLX1570 is an analog of b-AP15 that specifically inhibits USP14 and UCHL5 with improved solubility and potency [179]. It acts via the disruption of the ubiquitin proteasome degradation pathway, leading to an increase in poly-ubiquitinated proteins, inducible forms of chaperone Hsp70 and other markers characteristic of proteotoxic stress, ER stress, and oxidative stress leading to apoptosis [179,180].

Curcumin (diferuloylmethane): Curcumin is a natural polyphenolic compound extracted from the spice turmeric and reported to lead to ubiquitin proteasomal system dysregulation [181,182]. NF-κB p65 induces COP9 signalosome 5 (CSN5), required for PD-L1 stabilization via TNF-α in cancer cells. Curcumin also inhibits CSN5, diminishing PD-L1 expression in cancer cells and sensitizing cancer cells to anti-CTLA4 therapy [236]. In addition, curcumin exhibits anti-inflammatory, anti-oxidative, and anti-proliferative properties via the modulation of multiple cellular machineries [237].

AC17: AC17 is a 4-arylidene curcumin analog, developed as a 19S RP inhibitor, that acts as an irreversible USP14, UCHL5, and POH1 inhibitor, resulting in NF-κB pathway inhibition and reactivation of p53 [183]. AC17 causes a rapid and marked accumulation of poly-ubiquitinated proteins in A549 and NCl-H1299 cells, but the exact mechanism by which it induces the accumulation of ubiquitinated proteins is not known [183].

Δ12-PGJ2 and 15Δ-PGJ2: Δ12-PGJ2 and 15Δ-PGJ2 are inhibitors of the UCH family DUBs. Δ12-PGJ2 inhibits UCHL1 and UCHL3 with approximately 3.5 and 8.1 µM Ki, respectively, while 15Δ-PGJ2 only targets UCHL1 [184,185,186]. Δ12-PGJ2 has been reported to induce oxidative stress by causing a decrease in mitochondrial membrane potential, glutathione and glutathione peroxidases, and production of protein-bound lipid peroxidation products such as acrolein [184]. 15Δ-PGJ2 inhibits the activity of UCHL1 by affecting its structure and was the first protein reported to be oxidized in cells exposed to 15Δ-PGJ2 [185,186,187].

F6 (NSC 632839) and G5 compounds: These compounds were developed as broad-spectrum DUB inhibitors holding the same pharmacophore as Δ12-PGJ2 and induce apoptosis via accumulation of poly-ubiquitinated proteins [188]. G5 compounds induce apoptosis at low concentrations (~1 μM) and necrosis at higher concentrations (~10 μM) [190], whereas F6 inhibits the activity of USP2, USP7, and SENP2 deSUMOylase [189].

RA-9, RA-14, and AM146: These chalcone-based derivatives exhibit anti-cancer activities via DUB targeting without affecting the 20S proteasome catalytic core [191,192]. These inhibitors act on UCHL1, UCHL3, USP5, USP8, and USP2, which are known regulators of important cell survival and proliferation factors [191]. 

WP1130: WP1130 is the best-known USP9X inhibitor and has been reported to affect other DUBs (USP5, USP14, and UCHL5) [193,195]. WP1130 was identified in a screen for Janus kinase 2 (JAK2), and mass spectrometry shows a reversible covalent mechanism of action [196]. Inhibition of tumor-activated DUBs by WP1130 results in the downregulation of anti-apoptotic and upregulation of pro-apoptotic proteins such as MCL-1 and p53 and has also been reported to block the autophagic flux by ULK1 activity inhibition [193,194,195]. 

### 5.2. Other Small Molecule DUB Inhibitors

Numerous small molecules capable of inhibiting the activities of deubiquitinating enzymes have been investigated over the years. For example, USP7, or HAUSP, has been shown to have multifaceted roles in the cell cycle, DNA repair, chromatin remodeling, and epigenetic regulation. Many inhibitors have been developed to target USP7, such as P22077, HBX41108, HBX19818, HBX28258, P5091, and compound 14 [196,199,201,204,214,227]. 

Compound 14 and P22077: These compounds also inhibit USP47 along with USP7 [199,227]. Treatment of cells with Compound 14 leads to cell penetration and modulation of p53 and p21 as well as modestly accelerating polβ protein degradation in HeLa cells, confirming their role as dual USP7/USP47 inhibitors [199]. P22077 predominately inhibits USP7, showing an initial decrease in MDM2 levels followed by increased p53 and p21 levels, thereby regulating the p53 apoptotic pathway [227]. Orthotropic neuroblastoma mouse models showed an inhibition of xenograft growth in studies using P22077 [238]. P22077 also inhibits USP10 while playing a role in the degradation of oncogenic fms-related TK 3 (FLT3) kinase [197,198].

HBX family compounds: HBX41108 was the first sub-micromolar USP7 inhibitor reported but was later shown to be a non-specific DUB inhibitor [196,200]. HBX19818 and HBX28258 have been subsequently developed as more selective amidotetrahydroacridine derivatives, however, with lower potency [201]. HBX19818 shows preferential binding to the catalytic Cys residue of USP7 over other cysteinyl groups, stabilizing p53 and promoting G1 arrest and apoptosis in cells [201]. HBX19818 was recently reported to be a USP10 inhibitor that degrades spleen TKs and FLT3, resulting in the death of acute myeloid leukemia cells [202].

P5091: P5091, as with P22077, is another non-specific USP7 inhibitor from the Progenra’s thiophene chemical series [203,204]. P5091 stabilizes p53 and inhibits tumor growth in multiple myeloma cells and was found to be well tolerated in animal models while prolonging survival [204]. P5091 suppresses ovarian cancers harboring wild-type or mutant p53 genes and can effectively inhibit cell growth and induce both necrosis and apoptosis [239].

XL177A: XL177A is a selective (proteome-wide), highly potent (sub-nanomolar), and irreversible USP7 inhibitor. Transcriptome-wide analysis shows the specific upregulation of p53 transcriptional targets by XL177A [205]. Two pediatric cancers, malignant rhabdoid tumor (MRT) and Ewing sarcoma, that show sensitivity to other p53-dependent cytotoxic drugs, displayed more sensitivity to XL177A [205].

Other USP7 inhibitors: Almac Discovery and Genentech have reported fragment-based screening techniques that provide hits as starting points for USP7 discovery programs, and ADC-01 is one such hit. Utilizing X-ray crystallography techniques, they have produced a highly selective USP7 inhibitor called ADC-03 [206]. GNE-6640 and GNE-6776 are USP7 inhibitors discovered using nuclear magnetic resonance (NMR)-based screening. They enhance cytotoxicity and induce tumor cell death along with chemotherapeutic agents and targeted compounds such as PIM kinase inhibitors [207]. Simultaneously, another group has also identified two compounds, FT671 and FT827, as high-affinity USP7 inhibitors that target a dynamic pocket near the catalytic center of the auto-inhibited apo form of USP7 [208].

ML364, 5-(2-thienyl)-3-isoxazoles derivatives: USP2a is an important target for inhibition that could aid cancer therapy. ML364 has been identified as a potential small molecule inhibitor of USP2 with an IC_50_ of 1.1 µM, which induces cellular cyclin D1 degradation leading to cell-cycle arrest in HCT116 cancer cell lines and also has a lower affinity to USP8 [209]. ML364 selectively inhibits USP2 and thereby increases mitochondrial ROS levels, modulating the morphology and membrane potential of the mitochondria [240]. Two other small molecule inhibitors of USP2a, derivatives of 5-(2-thienyl)-3-isoxazoles, have been identified using NMR-based fragment screening and biophysical binding assays [210]. 

Vialinin A: Vialinin A, a non-specific USP4 inhibitor with an IC_50_ value of 1.5 µM, also targets the catalytic activity of USP5/isopeptidase T (isoT) and UCH-L1 [211]. Vialinin A plays a role in the prevention of VEGF-induced neovascularization, and research suggests that its natural anti-oxidative properties could be developed as anti-angiogenic agents in cancer therapy [212].

9-Oxo-9H-indeno[1,2-b]pyrazine-2,3-dicarbonitrile and analogs: 9-Oxo-9H-indeno[1,2-b]pyrazine-2,3-dicarbonitrile and its analogs have been identified by high-throughput screens as selective USP8 inhibitors that show anti-proliferative and pro-apoptotic activities in various cancer cell lines [213,214]. 9-Ethyloxyimino-9H-indeno [1,2-b]pyrazine-2,3-dicarbonitrile has been identified as an USP8 inhibitor that is structurally similar to USP7 inhibitors, and derivatives of these compounds have shown to be efficacious in mouse lung cancer models [213,214]. 

IU1 (1-[1-(4-fluorophenyl)-2,5-dimethylpyrrol-3-yl]-2-pyrrolidin-1-ylethanone): IU1 is an active-site-directed thiol protease small molecule inhibitor that selectively inhibits Usp14 [215]. It has been recently reported that IU1-mediated inhibition of USP14 results in the increased ubiquitination of constitutive photo-morphogenesis 9 (COP9) signalosome subunit 5 (COPS5), a key negative p53 regulator, resulting in tumor regression of autochthonous T-lymphomas and sarcomas in p53-deficient mice without affecting normal tissues [216]. 

[1,2,3] Triazolo [4,5-d] pyrimidine and derivatives: [1,2,3] Triazolo [4,5-d] pyrimidine and derivatives have been identified as inhibitors of USP28, especially compound 19, which has an IC_50_ value of 1.1 µM/L and a K_d_ value of 40 nM/L [217]. This novel inhibitor directly inhibits USP28 and induces the inhibition and degradation of the cell cycle, cell proliferation, and EMT progress in gastric cell lines [217].

3-Amino-2-keto-7H-thieno[2,3-b]pyridin-6-one derivative: 3-Amino-2-keto-7H-thieno[2,3-b]pyridin-6-one derivative is a UCHL1-targeting small molecule, discovered as a moderately potent non-competitive inhibitor that works by binding only to the Michaelis complex and not to free enzyme [218].

Isatin O-acyl oximes: Isatin O-acyl oximes are UCHL1-targeting inhibitors that can selectively inhibit UCHL1 in preference to its systemic isoform UCHL3 [219]. LDN57444 and LDN91946 are examples of UCHL1-inhibiting isatin O-acyl oximes [219]. Inhibition of UCHL1 activity using LDN5744 or its nanoparticle formulation LDN-POx in vitro shows inhibition of exosome secretions and reduced levels of pro-metastatic factors in exosomal fractions while suppressing the motility of metastatic squamous carcinoma cells and nasopharyngeal cells expressing EBV pro-metastatic latent membrane protein 1 (LMP1) [220]. LDN and LDN-POx treatment also showed decreased carcinoma cell adhesion, reduced pro-metastatic markers, and inhibition of extracellular vesicle-mediated transfer of invasive factor LMP1 [220].

Pimozide: Pimozide was initially developed as a selective USP1 inhibitor with sub-micromolar potency, shown to sensitize platinum-resistant NSCLC cells and promote PCNA and FANCD2 mono-ubiquitination [221]. Although pimozide studies show intended results, DUB selectivity profiling suggests non-specific behavior of the drug [196]. 

ML323: ML323 is another notable USP1/UAF1 complex inhibitor containing a selective pyrimidine core compound and has been shown to allosterically block UAF1 and USP1 complex formation, potentiate cisplatin cytotoxicity, and increase PCNA and FANCD2 mono-ubiquitination in cells [222,223]. However, not much progress has been made in advancing USP1 inhibitors to clinical settings. 

Mitoxantrone: Mitoxantrone was previously used for the treatment of acute myeloid leukemia, hormone-refractory prostate cancer, and multiple sclerosis and is the only reported topoisomerase inhibitor of USP11 that functions via an unknown mechanism [224]. 

Eeyarestatin-1 and GSK2643943A: Eeyarestatin-1 (Eer1) has been reported to specifically target DUBs associated with p97/VCP, such as Ataxin-3 [225]. A potent USP20 inhibitor identified by GSK, GSK2643943A, showed an IC_50_ of 160 nM [226].

## 6. Drug Development Challenges and Ongoing Clinical Trials 

Dramatic advancements in the field of UPS have greatly enhanced our understanding of the functions and mechanisms of this system. Previously, researchers focused on identifying compounds that disrupt E3 ligase and its substrate interactions, which can be intrinsically more difficult to achieve than searching for small molecule catalytic blockers. The challenges faced by researchers include identifying potent compounds that could selectively target DUBs at their catalytic pockets. The second challenge faced is due to Ub transfer via reactive thiol groups by DUBs, which interferes with the screening of DUB inhibitors. This is because the standard assays that have been used to identify inhibitors are limited by non-selective redox or alkylating false positives [241]. The complex mechanisms of DUBs as they alternate between active and non-active conformations also present a challenge when designing predictive biochemical assays and developing drug-like compounds [242,243]. Finally, the Ub specificity between the DUBs and the target proteins poses a challenge to optimizing the likelihood of identifying genuine inhibitors.

Targeting upstream regulators of the UPS, such as the E1, E2, and E3 enzymes, provides another level of control over the ubiquitination–deubiquitination system. Even targeting the proteasome has proven a successful clinical therapy, highlighted by the success of some FDA-approved inhibitors such as bortezomib [244], carfilzomib [245], oprozomib (ONX0912) [246], and ixazomib [247]. Inhibitors that have been identified to target the UPS and are under pre-clinical trials are summarized in Table 2. 

However, these drugs have shortcomings, such as fatigue, asthenia, drug resistance (bortezomib), and cardiovascular complications (carfilzomib), and need further optimization. Understanding the mechanism of E3 ligase specificity toward E2 enzymes and substrates and the trigger of the proteasomal degradation pathway due to lysine specificity could help explain the underlying mechanisms via structure–function studies. These avenues could open new targeting strategies for the development of highly specific and potent inhibitors. Despite the growing attractiveness of DUBs as therapeutic targets and the advancements in the field of DUB discovery and target identification, only a handful of DUB inhibitors, such as VLX1570, have advanced through clinical trials for cancer therapy. However, these phase I trials had to be prematurely terminated due to severe toxicity [284]. Complete information regarding clinical trials can be accessed using the clinical trial ID [285], and the promising DUB inhibitors undergoing clinical trials are reviewed in this section. 

The naturally occurring compound curcumin, which has been reported to have UPS dysregulation properties, has been extensively studied in clinical settings, especially in cancers [286]. Samsung Medical Center completed a placebo-controlled, double-blind, randomized trial in 107 male participants using curcumin in 2017 (NCT03211104). They aimed at establishing whether curcumin influenced the duration of treatment interruption and rate of prostatic specific antigen (PSA) progression, compared with placebo, among men with prostate cancer receiving intermittent androgen deprivation therapy. A phase II trial initiated by Emory University (NCT02944578) in 2016 is attempting to evaluate the biomolecular effects of curcumin capsules in high-grade squamous intraepithelial lesion (HSIL) cervical neoplasia. This drug is being tested in 40 women and has been postulated to affect cancerous cells by modulating various cellular pathways and altering the effect of HPV on tissue cells. This study aims at exploring the effect of curcumin as a potential medical treatment in HIV-infected women with HSIL lesions of the cervix. A recent study proposed by the Medical University of South Carolina (NCT03980509) in 2019 is recruiting to determine the effect of oral administration of curcumin in primary breast cancer tumors in 20 participants. This phase I trial will use oral administration of curcumin to check DNA-fragmentation-related apoptosis and cell proliferation (Ki67) in primary tumors of breast cancer patients.

Mitoxantrone is an FDA-approved drug that reportedly inhibits USP11. Numerous phase I/II clinical trials are currently being conducted using mitoxantrone to target various diseases, including neoplasms, relapsed/refractory acute myeloid leukemia, multiple sclerosis, advanced recurrent or metastatic breast cancer, neuromyelitis optica, and more [287,288]. The interventional study NCT02043756, completed in 2014, tested the pharmacokinetics, toxicity, and maximum tolerated dose of mitoxantrone hydrochloride (plm60-s) liposome injections in 20 participants with solid tumors. This study reported that a dose of up to 18 mg/m(2) of plm60-s had potential efficacy and was well tolerated [288]. A currently active phase II trial, initiated in December, 2018 is being conducted by CSPC ZhongQi Pharmaceutical Technology Co., Ltd. (NCT03776279). This trial aims to evaluate the safety and efficiency of mitoxantrone hydrochloride liposome injections in 106 participants with relapsed/refractory peripheral T-cell and NK/T-cell lymphoma. Michael Boyiadzis’ group, from the University of Pittsburgh (NCT03839446), initiated a phase II study in 2019 to examine the efficiency and toxicity of mitoxantrone in combination with etoposide and gemtuzumab ozogamicin (MEGO) in acute myeloid leukemia patients. This study is aimed towards patients who did not respond to first-line induction therapy to examine the efficacy and toxicity of this combinational therapy. 

Another important DUB inhibitor that has advanced to clinical trials is pimozide, which is currently in a phase II trial in patients with amyotrophic lateral sclerosis (ALS). The University of Calgary is actively conducting two clinical trials using pimozide (NCT02463825, NCT03272503). The first study, initiated in 2015, is a phase II trial that evaluates the effect of pimozide in 25 patients with neuromuscular junction transmission dysfunction due to amyotrophic lateral sclerosis (ALS) (NCT02463825). The second study, initiated in 2017, is a phase II placebo-controlled randomized trial using 100 ALS patients (NCT03272503). This study aims to test the effect of pimozide in slowing the progression of ALS. Successful clinical trials were also conducted in 2019 using betulinic acid, hypothesized to demonstrate anxiolytic and/or stress-reducing properties (NCT03904511). A betulinic-acid-containing plant extract called the Souroubea-Platanus preparation was administered to 45 healthy participants to study its safety, tolerability, and behavioral effects in healthy volunteers (NCT03904511). A complete list of inhibitors targeting DUBs and UPS enzyme components, along with their associated diseases, which are in different stages of clinical trials, is summarized in Table 3. 

## 7. Conclusions

The regulation of many critical proteins and cellular signaling events by DUBs modulates homeostasis and cell fate. Dramatic advancements in understanding of the ubiquitin system and identification of the crystal structures of various DUBs have paved the way for new possibilities in drug discovery and treatment of proteopathies, neurological disorders, and cancer. Parallel to the research focused on discovering different facets of DUB activity, the increasing number of inhibitors identified against this system have proved to be efficacious and selective in treating several disorders, including cancer. These inhibitors targeting the UPS system components are summarized in Table 3.

Despite our growing understanding of DUB biology, considerable work needs to be done to apply DUBs in clinical research. In-depth studies are therefore required to understand their natural regulatory mechanisms. In addition, identification of candidate pathways and targets in a cell that may assist DUB pharmacology also requires attention. Furthermore, pharmacodynamic effects of DUBs can be explored for possible avenues in clinical research. Besides DUB inhibitors, drugs enhancing DUB activities or expression should also be considered for further research. Another gray area in the field of DUBs is the number of targets regulated by a single DUB and vice versa. This raises a new challenge in itself as the inhibitors developed could have severe adverse effects by affecting non-target pathways. A combination of technologies, such as genomics, proteomics and structural analysis of DUBs, and an examination of their interactions with specific targets, combined with drug delivery strategies, could help advance the field of DUB inhibitors and accelerate them into the clinical setting. Combination therapies consisting of already approved proteasomal inhibitors along with novel DUB inhibitors could help abate the side effects of these drugs and also provide an interesting line of investigation in current research.

## Figures and Tables

**Table 1:** Summary of DUB inhibitors in oncology.

Inhibitor	DUB Target	Mechanism of Action	Reference
b-AP15	USP14, UCHL5	19S RP inhibitor	[177,178]
VLX1570	USP14, UCHL5	Induction of apoptosis	[179,180]
Curcumin	UPS dysregulation	CSN5 mediated PD-L1 inhibition	[181,182]
AC17	USP14, UCHL5, and POH1	19S RP inhibitor	[183]
Δ12-PGJ2	UCHL1, UCHL3	Oxidative stress	[184]
15Δ-PGJ2	UCHL1	Inhibition of hydrolase activity	[185,186,187]
F6 (NSC 632839)	USP2, USP7, and SENP2 deSUMOylase	Induction of apoptosis	[188,189]
G5 compounds	Broad-spectrum DUB inhibition	Induction of apoptosis	[190]
RA-9, RA-14, AM146	UCHL1, UCHL3, USP2, USP5, and USP8	DUB active site targeting	[191,192]
WP1130	USP9X, USP5, USP14, and UCHL5	DUB active site targeting	[193,194,195,196]
P022077	USP7, USP47, USP10	Induction of p53-mediated apoptosis	[197,198]
Compound14	USP7, USP47	Induction of p53-mediated apoptosis	[199]
HBX41108	USP7, Non-specific DUB inhibitor	Uncompetitive reversible p53-mediated inhibition	[196,200]
HBX19818	USP7, USP10	DUB active-site-targeted nucleophilic attacks	[201,202]
HBX28258	USP7	DUB active-site-targeted nucleophilic attacks	[201]
P5091	USP7	Cytotoxicity via HDM2-p21 signaling and p53	[203,204]
XL177A	USP7	Upregulation of p53 transcriptional targets	[205]
ADC-01, ADC-03	USP7	Unknown	[206]
GNE-6640, GNE- 6776	USP7	Attenuate Ub binding at catalytic cysteine	[207]
FT671, FT827	USP7	Target dynamic pocket at catalytic site	[208]
ML364	USP2, USP8	Reversible active site inhibition	[209]
5-(2-thienyl)-3-isoxazoles	USP2a	Inhibit catalytic binding site	[210]
Vialinin A	USP5/isopeptidase T (isoT) and UCH-L1	Competitive active site inhibition	[211,212]
9-Ethyloxyimino-9H-indeno[1,2-b]pyrazine-2,3-dicarbonitrile	USP8	Unknown	[213,214]
9-oxo-9 H-indeno[1,2-b]pyrazine-2,3-dicarbonitrile	USP8	O-alkyloxime moieties at position 9 of the tricyclic scaffold	[213,214]
IU1	USP14	Suppression of Ub chain trimming	[215,216]
[1,2,3] Triazolo [4,5-d] pyrimidine	USP28	Benzyl group attached to triazole ring required for its activity	[217]
3-Amino-2-keto-7H-thieno[2,3-b]pyridin-6- one derivative	UCHL1	Carboxylate group at 5-position and 6-pyridone ring responsible for inhibition	[218]
LDN57444, LDN91946	UCHL1	Reversible, active-site-directed inhibitors	[219,220]
Pimozide	USP1, Non-specific DUB inhibitor	Reversible, non-competitive inhibition	[196,221]
ML323	USP1/UAF1	Reversible, non-active-site-targeting inhibitor	[222,223]
Mitoxantrone	USP11	USP11 inhibition via unknown mechanism	[224]
Eeyarestatin-1	Ataxin-3	Unknown	[225]
GSK2643943A	USP20	Unknown	[226]
PR-619	Broad-spectrum DUB inhibitor	Accumulation of 26S proteasomal complexes	[227]
Betulinic acid	Broad-spectrum DUB inhibitor	Enhanced degradation of proliferation and pro-survival proteins	[228]
TCID	UCHL3	Unknown	[229]
EOAI3402143 (G9)	USP9X/USP24,USP5	Oxidative-stress-mediated apoptosis	[230,231]
Spautin-1	USP10, USP13	Inhibits autophagy via Beclin1 in Vps34 complexes	[232]
C527	USP1/UAF1	Degradation of ID1 causing p21 upregulation and cell cycle arrest	[233]
15-oxospiramilactone (S3)	USP30	Promotes mitochondrial fusion via Mfn1/2 ubiquitination	[234]

**Table 2:** Inhibitors of the UPS.

Inhibitor	Target	Reference
PYR-41	E1 enzyme	[248,249]
MLN7243	E1 enzyme	[250]
MLN4924	E1 enzyme	[251]
Compound 4b	E1 enzyme	[252]
PYZD-4409	E1 enzyme	[253]
Leucettamol A	E2 enzyme	[254]
Manadosterols A and B	E2 enzyme	[255]
CC0651	E2 enzyme	[256]
Nutlins and derivatives, RITA, MI-219, Syl-155, MI-63, PRIMA-1, HLI98, HLI373, MEL23 and MEL24, ATSP-7041, NSC207895	Mdm2/Mdmx/p53-mediated E3 ligase enzyme	[257,258,259,260,261,262,263,264,265,266,267]
Oridonin, SCF-12, ZL25, Compound A, Erioflorin, GS143, SMER3, TAME, Apcin	SCF E3 ligase	[268,269,270,271,272,273,274,275,276]
Bortezomib, CEP-18770, Carfilzomib (PR-171), ONX-0912, PR-047, MLN9708 and MLN2238 (Ixazomib), Marizomib (NPI-0052)	Proteasomal inhibitors	[277,278,279,280,281,282,283]

**Table 3:** Clinical status and disease association of UPS system inhibitors.

Inhibitor	Target	Clinical Status	Cancer Type and Disease	Reference/ Clinical Trial ID
b-AP15	USP14, UCHL5	Patent (ID: W0201305869)Preclinical	Acute myeloid leukemia, Multiple myeloma, Mantle cell lymphoma, Neuroblastoma, Prostate cancer, Colon cancer, Ovarian cancer, Breast cancer, Large B cell lymphoma	[177,245,289,290,291,292,293]
VLX1570	USP14, UCHL5	Phase I/II, prematurely ended	Acute myeloid leukemia, Multiple myeloma, Mantle cell lymphoma, Neuroblastoma, Prostate cancer, Colon cancer, Ovarian cancer, Breast cancer, Large B cell lymphoma	[294,295]
Curcumin	UPS dysregulation	Phase I/II, trials going	Prostate cancer, Lung cancer, Crohn’s disease, Colorectal cancer, Head and neck cancer, Breast cancer, Colonic cancer, Metastasis, Advanced cancers, Chronic obstructive pulmonary disease, Metabolic syndrome	[296,297]
WP1130	USP9X, USP5, USP14 and UCHL5	Patent (ID: W02012204527 A2) Preclinical	Acute myeloid leukemia, Prostate cancer, Colon cancer, Lung cancer, Hepatocellular carcinoma, Mesothelioma, Chronic myelogenous leukemia	[193,298,299,300]
Pimozide	USP1	Phase I/IV trials in schizophrenia Phase II trials in Amyotrophic Lateral Sclerosis (ALS)	Schizophrenia, Psychotic Disorders, Tourette Syndrome, Amyotrophic lateral sclerosis (ALS)	NCT02463825, NCT03272503, NCT00374244
Mitoxantrone	USP11	FDA approved	Acute myeloid leukemia, Neoplasms, Breast cancer, Acute myelogenous leukemia, Lymphoblastic lymphoma, Diffuse large B-cell lymphoma, Burkitt lymphoma/Leukemia	[288,301,302]
Betulinic acid	Broad spectrum DUB inhibitor	Phase I	Anxiety, Stress, Psychological disorders	NCT03904511
MLN7243	E1 enzyme	Phase I	Advanced malignant solid tumors, Myelodysplastic syndrome, Recurrent/refractory acute myeloid leukemia, Refractory chronic myelomonocytic leukemia, Refractory high-risk myelodysplastic syndrome	NCT03816319
MLN4924	E1 enzyme	Phase I, completed	Acute myelogenous leukemia	[303,304]
Compound 4b	E1 enzyme	Phase I, completed	Neoplasms, Epilepsy, HIV infections	[305]
Bortezomib	Proteasomal inhibitor	FDA approved	Multiple myeloma, Mantle cell lymphoma, Leukemia, Neuroblastoma, Head and neck cancer, Thyroid carcinoma, Hepatocellular carcinoma, Amyloidosis, Cold agglutinin disease, Hemolytic anemia	[306,307,308,309,310,311]
Carfilzomib	Proteasomal inhibitor	FDA approved	Multiple myeloma, Relapsed and/or refractory multiple myeloma, Lymphoma, Leukemia Lung cancer, Thyroid carcinoma, Refractory renal cell carcinoma, Pulmonary hypertension	[312,313,314,315]
Ixazomib	Proteasomal inhibitor	FDA approved	Multiple myeloma, Relapsed and/or refractory multiple myeloma, Lymphoma, Leukemia, Breast cancer, Glioblastoma, Renal cell carcinoma, Bladder cancer, Hodgkin and T cell lymphoma, HIV, Lupus, Kidney diseases	[316,317,318,319]
Marizomib	Proteasomal inhibitor	Phase I/II/III ongoing	Multiple myeloma, Relapsed and/or refractory multiple myeloma, Lymphoma, Glioblastoma, Pancreatic cancer, Melanoma,	[320,321,322]
ONX-0912	Proteasomal inhibitor	Phase I/II, completed	Multiple myeloma, Relapsed and/or refractory multiple myeloma, Hepatocellular carcinoma, Non-central-nervous-system malignancies	[323,324,325]

## Abbreviations

DUBs	Deubiquitinating enzymes
UPS	Ubiquitin proteasome system
UCHs	Ubiquitin C-terminal hydrolases
OTUs	Ovarian tumor proteases
MJDs	Josephin or Machado–Joseph disease protein domain proteases
JAMM	Jab1/MPN domain-associated metalloisopeptidase domain proteins
MCPIP	Monocyte chemotactic protein-induced protein family
MINDY	Motif interacting with Ub-containing novel DUB family
ZUFSPs	Zn-finger and UFSP domain proteins
GWAS	Genome-wide association studies
RTKs	Receptor tyrosine kinases
Ub	Ubiquitin
BAP1	BRCA1-associated protein 1
MM	Multiple myelomas
MCL	Myeloid cell leukemia
CDH11	Cadherin11 gene
NF-κB	Nuclear factor-κB
IKK	IκB kinase
TPA	12-O-tetradecanoylphorbol-13 acetate
TNFAIP3	tumor necrosis factor, alpha-induced protein 3
HCF-1	Host cell factor-1
TGF-β	Transforming growth factor-β
AIF	Apoptosis-inducing factor
FASN	Fatty acid synthase
EGFR	Epidermal growth factor receptor
MEFs	Mouse epithelial fibroblasts
FOXO4	Forkhead box protein O4
PTEN	Phosphatase and tensin homolog
NSCLC	Non-small-cell lung cancer
LSD1	Lysine-specific demethylase1
TP53BP1	TP53-binding protein 1
MCPIP1	MCP-induced protein 1
TK	tyrosine kinase
PCP	Planar cell polarity
EMT	epithelial-to-mesenchymal transition
R-SMAD	Receptor-regulated SMADs
DDR	DNA damage response
19S RP	19S regulatory particle
CSN5	COP9 signalosome 5
JAK2	Janus kinase 2
FLT3	Fms-related tyrosine kinase 3
CYLD	Cylindromatosis
APC	Anaphase-promoting complex
Plk1	polo-like kinase 1
